# Spin Seebeck effect mediated reversal of vortex-Nernst effect in superconductor-ferromagnet bilayers

**DOI:** 10.1038/s41598-023-31420-2

**Published:** 2023-03-17

**Authors:** Himanshu Sharma, Zhenchao Wen, Masaki Mizuguchi

**Affiliations:** 1 Graduate School of Engineering, Nagoya University, Nagoya, 464-8603 Japan; 2 CREST, Japan Science and Technology Agency, Kawaguchi, 332-0012 Japan; 3 Imec, Kapeldreef 75, 3001 Leuven, Belgium; 4 Department of Chemistry, KU Leuven, Celestijnenlaan 200F, 2404, 3001 Leuven, Belgium; 5 National Institute for Materials Science (NIMS), Tsukuba, Ibaraki 305-0047 Japan; 6 Center for Spintronics Research Network, Osaka University, Toyonaka, 560-8531 Japan

**Keywords:** Energy science and technology, Engineering, Materials science, Nanoscience and technology, Physics

## Abstract

We report on the observation of sign reversal of vortex-Nernst effect in epitaxial NbN/Fe bilayers deposited on MgO (001) substrates. Strong coupling between vortex magnetisation and ferromagnetic magnetisation at the NbN/Fe bilayer interface is presented. In NbN/Fe bilayer thin films an apparent sign reversal of vortex-Nernst signal under a temperature gradient with magnetic field and temperature is observed when the thickness of Fe is increased up to 5 nm. This reversal of the vortex-Nernst effect is associated with the enhancement of the spin Seebeck effects (SSE) near *T*_c_ due to coherence peak effect (CPE) and strong coupling of vortex magnetisation and ferromagnetic magnetisation at the interface of the NbN/Fe bilayer. The observed large SSE via inverse spin Hall effect (ISHE) is due to the CPE below and close to *T*_*C*_, highlighting the high spin to charge conversion efficiency of NbN in this region. This work may contribute to the development of superconducting spintronic devices by engineering the coupling of the superconductor/ferromagnet interface.

## Introduction

**Figure 1:**
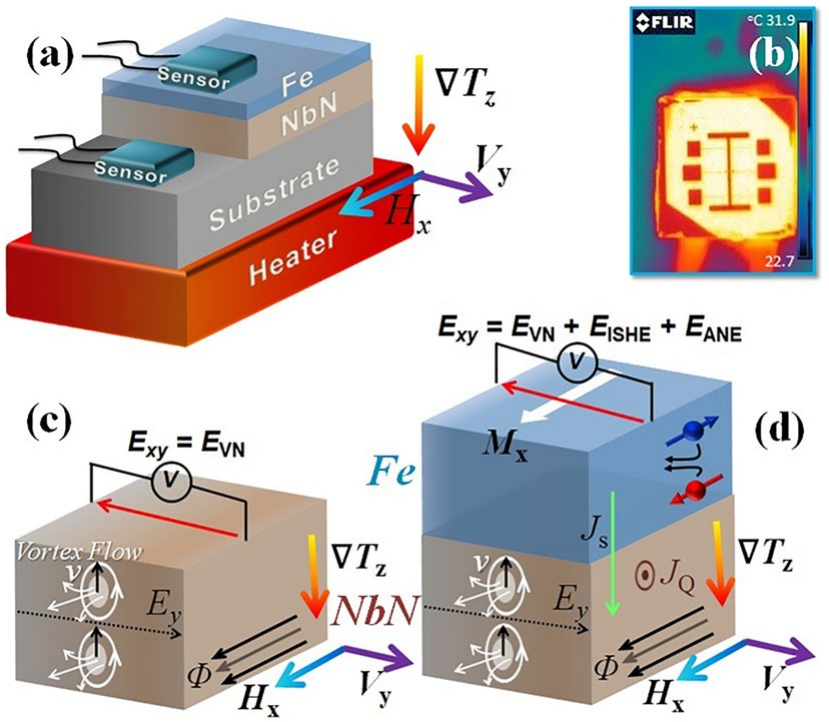
(**a**) Schematic of setup for thermally induced transverse voltage measurements, (**b**) Thermo-graphic image of a device with heater. Schematic configurations for (**c**) vortex Nernst effect and (**d**) transverse voltage generated in NbN/Fe bilayer.

Superconducting spintronics is a promising field that combines spintronics, superconductivity and magnetism, offering new opportunities to procure spin transport with minimal Joule heating and dissipation energy^1–6^. However, the unusually weak coupling^1^ of spin and charge transport due to the spin-singlet condensate in *s*-wave and *d*-wave superconductors is a major challenge in this field. To date, the formation of spin-triplet condensate due to the proximity effect^6–12^ and thermally excited quasiparticles (QPs)^13–16^ can carry spin angular momenta at superconductor-ferromagnet (SC/FM) interfaces, which has provided a possibility for the interaction between superconductivity and spin-polarization^6–16^. On the other hand, spin-caloritronics^16–27^ with superconductors^2,13,14,27–37^ is an emerging field where several physical parameters, viz. charge, heat, QPs, vortices, spin and spin current, excited by thermal gradients interact simultaneously to induce various physical phenomena such as, the spin Nernst effect (SNE)^17,18^, the Nernst effect/anomalous Nernst effect (ANE)^19–26^, the spin Seebeck effect (SSE)^13,18,27^, the inverse spin Hall effect (ISHE)^2,13,15,18^, the vortex Nernst effect (VNE)^13,27–37^, etc. In high-temperature superconductors (HTS), the Nernst effect has attracted much attention due to the discovery of a sizable Nernst coefficient because of superconducting fluctuations^27–37^. Previous studies have apparently explained how Gaussian superconducting fluctuations due to fluctuating Cooper pairs^29,32^, quasi-particles^31^, and superconducting vortices^29–37^ play a key role in the generation of a large Nernst signal in high-temperature type-II superconductors. Below the superconducting transition temperature (*T*_*c*_), the Nernst signal^28–30^ is generated by the long-lived vortices of the vortex liquid^29^, which is known as the VNE. However, above *T*_*c*_ but within the transition region, Cooper-pair fluctuations are mainly responsible for the sizable Nernst signal^28^. In addition, ISHE, where a spin current due to SSE can be converted into a charge current by the Onsager reciprocal relation, has attracted much attention in HTS. This is because of the discovery of a surprisingly large ISHE signal due to the QPs by electrical spin injection in HTS (Fig. 1)^2,13^.

Recently, an enhancement in ISHE induced by spin injection from a ferrimagnetic insulator into a superconductor using longitudinal spin Seebeck effects (LSSE) has been observed in a narrow temperature range just below *T*_*c*_^13^. This enhancement of ISHE is associated with the increase in spin dynamics due to the singularity in the QP density of states near *T*_*c*_ in full-gap superconductors, which is known as coherence peak effects (CPE)^13^. This research demonstrates that ISHE can be used as a technique to reveal coherence effects in superconductors.

However, in the field of spin-caloritronics with SC/FM system^33,34^, the effect of SC/FM interfaces on the vortex Nernst effect and ISHE is still unclear. The study of such thermally induced spin-caloritronic phenomena^6,33–37^ at SC/FM interfaces not only facilitates spontaneous discoveries, but also provides an insight into superconducting spintronics.

In this paper, we present a comprehensive investigation of the VNE in NbN (20 nm) and bilayers of NbN (20 nm)/Fe (*t* nm) for different values of *t* with out-of-plane thermal gradient and in-plane magnetic field below and near *T*_*c*_. In addition, we investigate the ISHE induced by spin injection from a ferromagnet (FM) into a superconductor in NbN (20 nm)/Fe (*t* nm) bilayers using LSSE. Structural, transport and magnetic properties of the NbN thin film and the bilayers of NbN/Fe thin films are also characterised for comparative studies.

## Results and discussion

**Figure 2:**
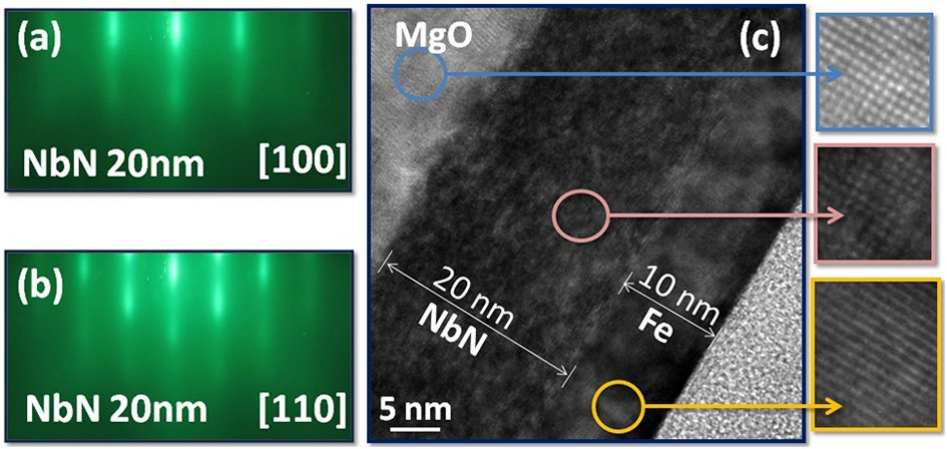
RHEED patterns of the NbN (20 nm) thin film surface imaged along the (**a**) [100] and (**b**) [110] directions, (**c**) HR-TEM image of NbN (20 nm)/Fe (10 nm) bilayer thin film sample. Insets highlight the alignment of lattices.

Figures 2a,b show real-time and in-situ reflection high-energy electron diffraction (RHEED) patterns in two diffraction directions [100] and [110] of the NbN (20 nm) thin film. Streaks in the RHEED pattern confirm the flat surface and epitaxial growth of the NbN thin film. The high crystalline quality and actual film thickness were also confirmed by a cross-sectional high-resolution TEM (HR-TEM) image of the NbN (20 nm)/Fe (10 nm) bilayer thin film, as shown in Fig. 2c. The HR-TEM image shows good lattice matching with the substrate along with clear and well-defined interface between NbN-Fe as well as MgO substrate-NbN layer. The insets of Fig. 2c highlight the lattice orientation, confirming the epitaxial growth of NbN with long-range cubic structure.

**Figure 3:**
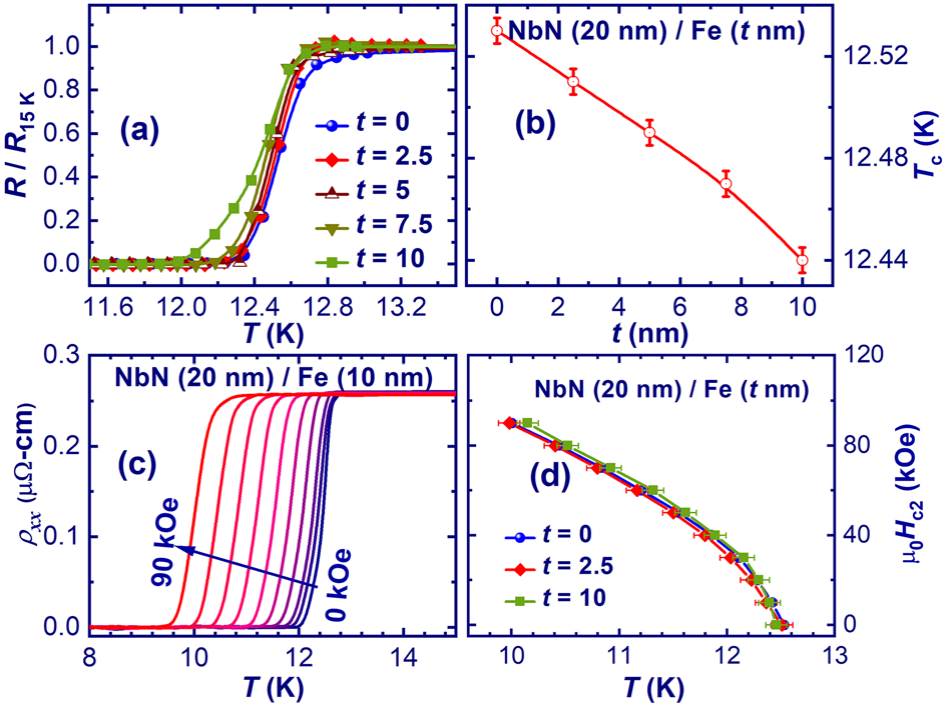
(**a**) Normalized resistance vs *T* of bilayer thin films with different Fe thickness (*t*) in zero magnetic field, (**b**) Variation of resistively measured *T*_*c*_ vs *t*, (**c**) *ρ*_*xx*_ vs *T* for NbN/Fe (10 nm) thin film with different applied magnetic fields ranging from 0 to 90 kOe with a step of 10 kOe, and (**d**) Upper critical field (*H*_*c2*_) vs *T* for NbN, NbN/Fe (2.5 nm) and NbN/Fe (10 nm) thin films, respectively. Solid lines are fit to GL model.

The transport properties of NbN thin film and NbN/Fe bilayer thin films with different Fe layer thicknesses were measured using a four-probe method. Figure 3a shows the normalised resistance (*R*/*R*_15 K_) as a function of temperature (*T*) to highlight the shift in the critical temperature (*T*_*c*_) of the bilayer thin films with different Fe thicknesses at zero applied magnetic field. This allows the variation of the resistively measured *T*_*c*_ to be plotted as a function of *t*, as shown in Fig. 3b. The decrease in *T*_*c*_ with the increasing Fe thickness clearly confirms the influence of the Fe layer on the superconductivity of the NbN layer.

In addition, we have measured the longitudinal resistivity (*ρ*_*xx*_) as a function of temperature for NbN/Fe (*t* nm) bilayer thin films at different in-plane applied magnetic fields. Figure 3c shows a set of *ρ*_*xx*_–*T* curves for NbN/Fe (10 nm) bilayer thin film measured at the magnetic fields ranging from 0 to 90 kOe, highlighting the magnetic response of the bilayers. We have also estimated the resistively measured upper critical field [*H*_c2_(*T*)], below which vortices appear in the superconducting sample. This is considered to be equal to the vortex-solid melting field (*H*_vs_). Figure 3d shows the upper critical field (*H*_c2_) with *T* for the NbN thin film, NbN/Fe (2.5 nm) and NbN/Fe (10 nm) bilayer thin films, respectively. Here, we have calculated the *H*_c2_ of the NbN thin film and NbN/Fe (*t* nm) bilayer at different value of *t* using, (i) the 50% criteria of the normal state resistivity (i.e. where the resistivity becomes 50% of its normal state value) and (ii) the derivative of the resistivity, at different applied magnetic fields to plot it with error bars. This *H*_c2_(*T*) approximation agrees well with the Ginzburg–Landau (GL) model^38^, which can be described as:

1 $${H}_{c2}(T)={H}_{0}[(1-{\tau }^{2})/(1+{\tau }^{2})]$$

where $\tau$ =*T*/*T*_*c*_, and ${H}_{0}$ is critical field at 0 K. Solid lines in Fig. 3d show the fit to the GL model.

The *H*_c2_ decreases with temperature for all samples and increases slightly with the thickness of the Fe layer. This indicates that the vortex-solid melting field modulates with varying Fe thickness. This small shift in *H*_c2_ could be related to the variation of interface transparency (i.e. a parameter of the proximity effect theory) with Fe thickness on the upper critical magnetic fields, consistent with previous work^9^.

In the case of NbN/Fe (10 nm) bilayer thin film, a crossover at low *H*_c2_ is observed due to inhomogeneous superconductivity associated with proximity effect at the NbN/Fe interface^39^.

**Figure 4:**
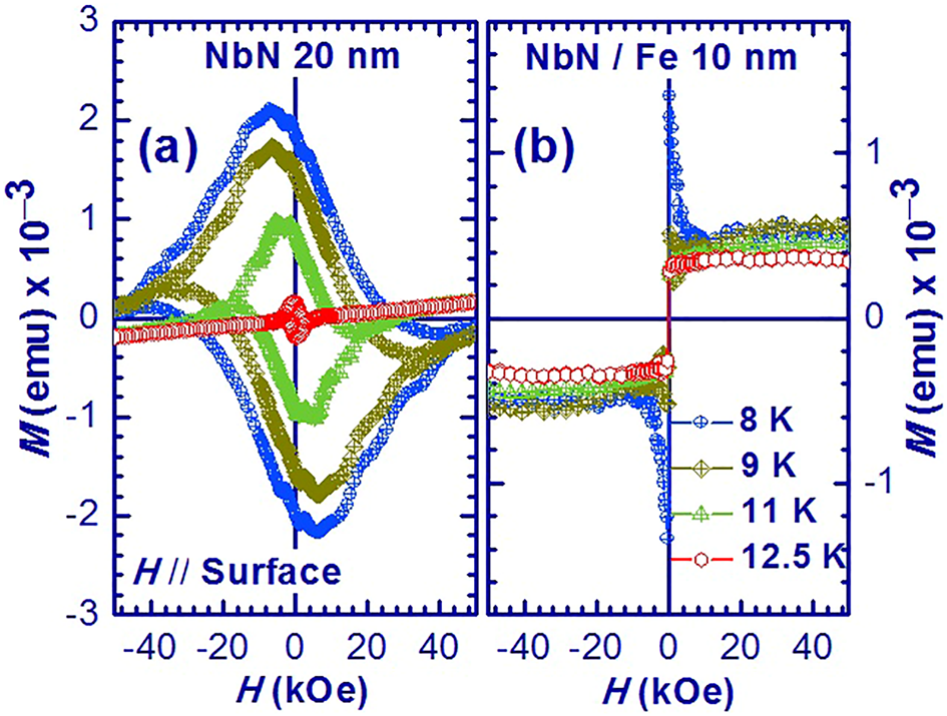
Magnetisation of (**a**) NbN (20 nm) thin film and (**b**) NbN(20 nm)/Fe(10 nm) bilayer thin film as a function of applied magnetic field at different temperatures.

Subsequently, we measured the in-plane magnetisation for the NbN thin film and the NbN/Fe (10 nm) bilayer thin film as a function of the applied magnetic field at different temperatures, as shown in Fig. 4a,b. The *M*-*H* loops for the NbN thin film show the expected type-II superconductor characteristics^33,40^ for *T* ≤ *T*_*c*_. The width of the *M*-*H* loops decreases significantly with increasing temperature from 8 to 12.5 K, which is associated with the expected decrease in vortex pinning strength^33,40^ in the single NbN thin film. On the other hand, the *M*-*H* loop of the NbN/Fe (10 nm) bilayer at 12.5 K (near *T*_*C*_), reflects the feature of ferromagnetic magnetisation of the top Fe layer. Furthermore, the magnetisation curves change dramatically below *T*_*C*_ and show irreversible behaviour due to the vortex magnetisation in the NbN layer. This irreversible behaviour observed in the *M*-*H* loops of NbN/Fe below *T*_*C*_ due to the increase in vortex pinning strength of NbN layer further confirms the coupling between the ferromagnetic magnetisation in the Fe layer and the vortex magnetisation in the NbN layer^6^.

**Figure 5:**
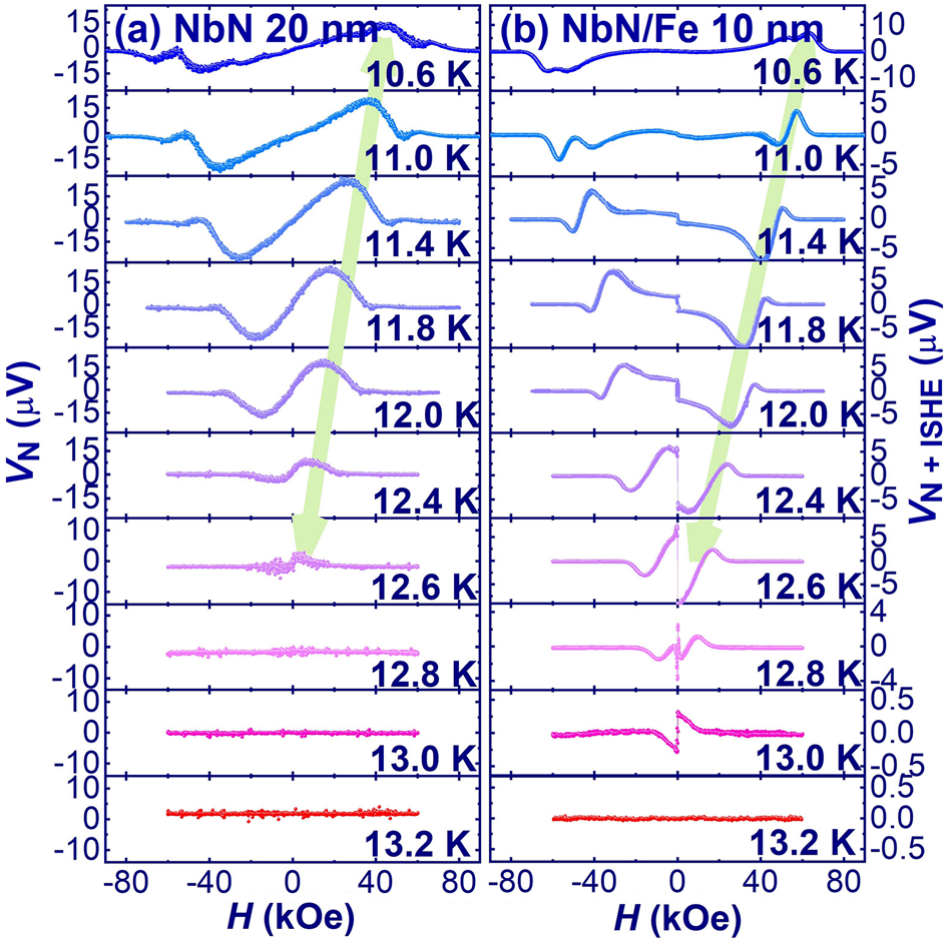
(**a**) VNE (*V*_*VN*_) vs *H* for NbN thin film of 20 nm and (**b**) thermally induced transverse signal (*V*_VN + ISHE_) in bilayer of NbN (20 nm)/Fe (10 nm) to highlight the behavior of VNE and ISHE at different temperatures.

Next, we measured the thermally induced transverse voltage which is the vortex-Nernst signal (VNE) *V*_VN_ as a function of applied magnetic field below *T*_*c*_ for NbN thin film with out of plane applied thermal gradient and in plane applied magnetic field as shown in Fig. 1c. For the type-II superconductor, the VNE (*V*_VN_) below *T*_*c*_ is generated by the long-lived movable vortices of the vortex liquid state, which is strongly nonlinear with the applied magnetic field^35–37^. In the steady state, the frictional force due to the moving vortices balances the thermal force exerted by the thermal gradient^35^. Thus, the VNE can be described phenomenologically as^35^:

2 $${V}_{VN}=\frac{H {S}_{\varnothing }}{\eta }=\frac{\rho {S}_{\varnothing }}{{\varnothing }_{0}}$$

where $\eta$ is the damping viscosity derived from the flux-flow resistivity ($\rho =H {\varnothing }_{0}/ \eta )$, ${\varnothing }_{0}=h/2e$ is the superconducting flux quantum and ${S}_{\varnothing }$ is the entropy transported per vortex^35^. Figure 5a shows the magnetic field dependence of the VNE for the NbN thin film at different temperatures. The vortex-induced Nernst signal shows a characteristic hill-shaped profile with a maximum at the magnetic field (*H*_*Max*_) for the NbN thin film. The magnitude of *H*_*Max*_ decreases with increasing temperature, which is consistent with the previous observation of VNE in type-II superconductors^35–37^.

On the other hand, the thermally induced transverse voltage in NbN/Fe bilayers is an addition of VNE (*V*_VN_) due to NbN, ISHE generated by LSSE and ANE due to top Fe layer as shown in Fig. 5b. Here, the nearly zero signal at temperature > 13 K (> T_c_) and nearly zero signal at the high magnetic field (> *H*_c2_) indicate the negligible contribution of ANE due to the top Fe layer at low temperature.

The thermally induced spin current (*J*_S_) from the top Fe layer injected into the NbN thin film propagates through the ISHE due to LSSE and converted into a charge current (*J*_c_). The generated charge current, a spin of an electron (or quasiparticle in the superconducting state) and the injected spin current are related as^2,18^:

3 $${J}_{C}\propto {J}_{S} \times s$$

where *J*_C_, *J*_s_ and s denote the charge current vector, the spin current vector and the spin-polarization vector of the spin current, respectively. Here, ${J}_{S}\propto {\mathrm{g}}_{\mathrm{ef}}^{\uparrow \downarrow }$, where ${\mathrm{g}}_{\mathrm{ef}}^{\uparrow \downarrow }$ is the spin-mixing conductance of the FM/SC interface and depends on the Gilbert damping of FM^2,18^. Furthermore, *s* is parallel to the magnetisation *M* of the Fe thin film as the spin current is generated from the Fe thin film (injector). In the superconducting state, the charge current *J*_C_ is substituted by the quasiparticle current *J*_Q_.

In single NbN thin film, the VNE increases with magnetic field due to the moveable nature of the vortices until the vortex solid state melts (see Fig. 5a). It reaches a maximum at *H*_*Max*_ and starts to decrease at *H* > *H*_*Max*_ due to the drop of the excess entropy of the vortex core (see Fig. 5a). The NbN/Fe bilayers also shows the similar characteristic hill-like profile at 10.2 K (i.e. far below *T*_c_) with an almost flat line at low magnetic fields as shown in Fig. 5b. This indicates the dominant nature of the VNE due to the enhancement of the vortex liquid state at low temperatures and almost zero ISHE. On the other hand, by further increasing the temperature, we can clearly see the negative ISHE signal due to thermally excited quasiparticles at low magnetic fields along with a fluctuating characteristic hill-like profile due to VNE at high magnetic fields. As the temperature approaches to *T*_c_, the ISHE increases and starts to dominate as the VNE decreases due to the drop of the excess entropy of the vortex core. In addition, *H*_*Max*_ also decreases with increasing temperature. This dominant nature of ISHE causes the absolute reversal of this hill-like profile of VNE near *T*_c_ in the NbN/Fe (10 nm) bilayer thin film, as shown in Fig. 5b. This increase in ISHE induced by SSE in bilayer films in a narrow temperature range immediately below *T*_c_ is consistent with that recently observed by Umeda et al. in the ferrimagnetic insulator Y_3_Fe_5_O_12_ (YIG) and NbN bilayer^13^. However, above *T*_*c*_ but within the transition region, the thermal excitation of the quasiparticle states increases further and initiates the braking of Cooper pairs, reflecting Cooper-pair fluctuations in this region. This Cooper-pair fluctuations may be responsible for the small positive ISHE signal at 13 K (see Fig. 5b). Further, above *T*_*c*_, as NbN approaches towards the normal state, the conductivity of NbN decreases and thus the spin-current leakage through the metallic FM starts, which in turn reduces the ISHE signal in the normal state.

**Figure 6:**
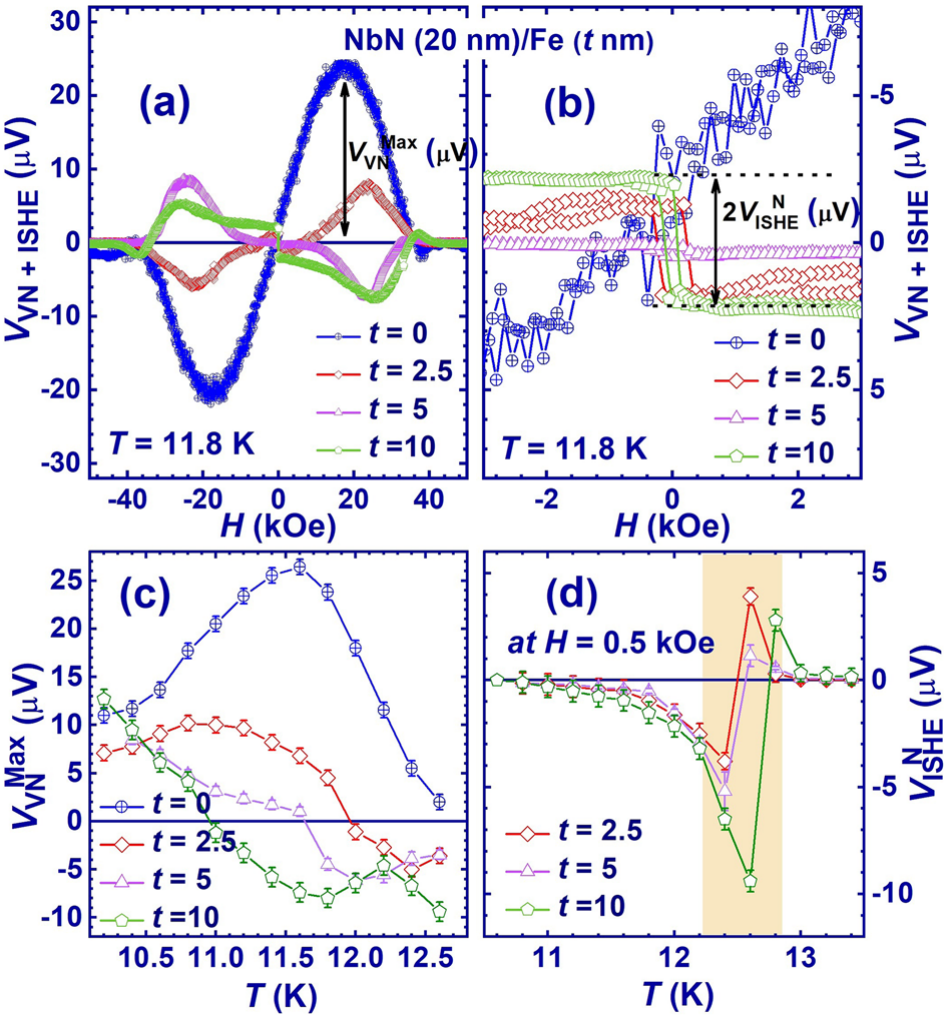
(**a**) Thermally induced transverse signal (*V*_VN + ISHE_) vs *H* for NbN thin film of 20 nm, bilayer thin films of NbN (20 nm)/Fe (*t* nm) with *t* = 2.5 nm, 5 nm, and 10 nm, (**b**) zoom to highlight ISHE signal (*V*_ISHE_) at low magnetic fields (**c**) ${V}_{VN}^{Max}$ as a function of temperature with different values of *t*, (**d**) variation of *V*_ISHE_ vs *T* with different values of thickness of Fe layer.

Further, to investigate the effect of film the thickness of the top ferromagnetic (Fe thin film) layer on the thermally induced transverse signal (*V*_VN + ISHE_), the *V*_VN + ISHE_ was measured as a function of applied magnetic field near *T*_*c*_ (below and above) for different thicknesses of the Fe layer. Figure 6a shows the *V*_VN + ISHE_ as a function of applied magnetic field for the NbN (20 nm)/Fe (*t* nm) bilayer thin films with *t* = 2.5 nm, 5 nm, and 10 nm below *T*_*c*_ (at 11.8 K). A *V*_VN_ vs. *H* curve of the NbN thin film (for *t* = 0 nm) is also plotted (in blue) to highlight the VNE of the NbN thin film. It is worth highlighting that the observed ISHE has a negative sign below *T*_*c*_ (at 11.8 K) for *t* > 0, compared to that of the VNE peak (see Fig. 6a,b) for *t* = 0. This agrees well with the results reported in previous research^13^. Fig. 6b confirms the variation of the amplitude of the ISHE signal with different value of *t.* The variation of *t* also has significant effect on the VNE. For *t* = 2.5 nm, the sign of the VNE is the same as that of the NbN thin film (see Fig. 6a). As the thickness of the top Fe thin film increases, a complete reversal of the VNE is observed, as shown in Fig. 6a. This reversal of VNE may be associated to the increase in ISHE with increasing Fe thin film thickness. Here, the peak of VNE as shown in Fig. 6a is the maximum vortex Nernst signal (${V}_{VN}^{Max}$), and ${V}_{ISHE}^{N}$ is the normalized ISHE signal as shown in Fig. 6b. Next, the peak of VNE, i.e. the maximum vortex Nernst signal (${V}_{VN}^{Max}$), is plotted as a function of temperature to see the variation of VNE with temperatures for different values of *t*, as shown in Fig. 6c. A clear reversal of the VNE can be seen in Fig. 6c. The variation of the ISHE signal as a function of temperature at an applied magnetic field of 0.5 kOe with different values of *t* is plotted in Fig. 6d. Here, we see a large increase in ISHE near *T*_c_, confirming the previously reported^13^ coherence peak effect (CPE) in the spin-Seebeck effects for superconductors in NbN/Fe bilayers.

Finally, we discuss the mechanism of the sign reversal of the vortex-Nernst (VNE) signal in the NbN/Fe bilayer films. The variation of this reversal behaviour of the VNE can be realised as a function of magnetic field and temperature by varying the thickness of the top Fe thin film. The VNE observed in the single thin film of NbN shows the hill-like profile due to the frictional force of freely moving vortices in the vortex liquid state, which diffuses from the hot to the cold end of NbN and generates a voltage along *y*-direction while moving along the *x*-direction^35–37^.

In contrast, in NbN/Fe, local magnetisation inhomogeneity develops due to complex polarisation and interfacial spin states of the NbN/Fe interface. It should be noted that certain forms of magnetic inhomogeneity at the interface of diffusive superconducting structures with a ferromagnet can lead to the formation of a triplet condensate, as reported elsewhere^41,42^. This is one of the possible mechanisms for the formation of the triplet condensate in the NbN/Fe bilayer.

Furthermore, thermally induced spin current flows across the interface of SC and FM layers due to spin-polarized QPs in SC and magnon excitation in FM through spin Seebeck effects (SSE). These spin-polarized QPs in SC are deflected by spin–orbit scattering and derive the spin-flip scattering of QPs in the SC layer at the interface by interacting with localised moments in the FM layer, which are linked with a magnon excitation in the FM layer^13^.

These low frequency magnons are responsible for the anomalous enhancement of the SSE near *T*_c_ due to CPE in superconductors^13^. The observed SSE, in the case of the bilayer follows the spin polarization at the interface and is consistent with the magnetisation measurement at low magnetic fields as shown in Fig. 4b (low field data at 12.5 K) and 5b (at 12.4 and 12.6 K). This indicates and confirms the strong coupling between the vortex magnetisation and the ferromagnetic magnetisation at the interface of the NbN/Fe bilayer.

The increased local ferromagnetic moments enhance the magnon excitation in the FM layer and hence the spin-flip scattering of QPs in the SC layer. These complex polarization and interfacial spin states can alter the motion of free-moving vortices in the vortex liquid state, resulting in a reversal of the VNE signal. Recently, Shuang Wu, et. al. reported that the Gilbert damping of Fe thin films increases below a critical thickness (~ 5 nm)^43^. This increase in Gilbert damping in Fe ultra-thin films decreases the spin-mixing conductance (${\mathrm{g}}_{\mathrm{ef}}^{\uparrow \downarrow }$) and hence the ISHE. Consequently, the VNE in the NbN (20 nm)/Fe (2.5 nm) bilayer shows a dominant nature of the vortex magnetisation, where a consistent (as in NbN) hill-shape profile was observed regardless of the hysteresis at low magnetic field (see Fig. 6a,b). While increasing the thickness of the FM layer enhances the spin-mixing conductance, localised ferromagnetic moments and its associated magnon excitation in the FM layer. This enhances the spin Seebeck effects near *T*_c_ due to CPE. This is further consistent with the observation of low CPE at lower temperature in NbN (20 nm)/Fe (2.5 nm) and NbN (20 nm)/Fe (5 nm). This work will bring an interesting aspect regarding the interplay between ISHE and vortex Nernst effect to create superconducting spintronic devices.

Concerning the origin of the thermally induced transverse voltage, we emphasised that it comes from VNE and ISHE, while the ANE from the Fe layer at low temperature is almost zero. This may be due to the low thermal gradient and very small ANE coefficient of Fe at low temperature. As shown in Fig. 5b, the nearly zero transverse signal at 13.2 K (*T* > *T*_*c*_) and the nearly zero transverse signal at the high magnetic field (> *H*_c2_) for all temperatures indicate the negligible contribution of ANE due to the top Fe layer at low temperatures. This confirms that ANE has no contribution to the thermally induced transverse voltage in the non-superconducting state (*T* > *T*_*c*_) of NbN. We take into account that in the FM layer (i.e., Fe thin film) ANE is generated in the form of direct transverse voltage and thus does not require spin to charge conversion material as in SSE. Therefore, ANE should persist in the non-superconducting state of NbN and should become more negligible in the superconducting state due to the increasing conductance of NbN.

However, ANE in Fe thin films is strongly dependent on the thickness of the Fe film, increasing with decreasing thickness and even changing sign below a critical thickness at room temperature^44^. Thus, the fact that ANE of Fe may possibly affect the properties of the vortex states and the voltage of the VNE, cannot be denied and remains to be investigated in future work.

## Conclusions

In conclusion, we have fabricated epitaxial NbN and NbN/Fe thin films and presented the sign reversal of the VNE in the NbN/Fe bilayers which can be realised as a function of magnetic field and temperature by varying the thickness of the top Fe thin film. The enhancement of the SSE near *T*_c_ due to CPE and the strong coupling of vortex magnetisation and ferromagnetic magnetisation at the interface of the NbN/Fe bilayer could contribute to the reversal of the vortex-Nernst effect. The first report of the interaction between the vortex state and the spin Seebeck effect (SSE) is presented. These results may provide a pathway for the development of superconducting spintronic devices using SC/FM interfaces.

## Materials and methods

The bilayer thin films of NbN (20 nm)/Fe (*t* nm) and a single thin film of NbN (20 nm) were deposited by dc-magnetron sputtering. The NbN thin film of preset thickness (20 nm) was deposited by sputtering a Nb-target in an Ar-N_2_ gas mixture (10:1) on MgO (001) substrates. To measure the longitudinal (*R*_*xx*_) and transverse (*R*_*xy*_) resistances, all the thin films were converted into patterned Hall bar structures by using the optical lithography and ion milling process, as shown in Fig. 1b. The typical lateral channel size of the Hall bars is 50 μm × 3 mm. The magnetic properties of NbN/Fe bilayers and NbN thin films as a function of temperature and magnetic field were measured using SQUID-MPMS. Transport measurements were carried out using PPMS. For thermoelectric measurements, i.e. Nernst effect, the Hall-bar structure was mounted on a platform to obtain the required out-of-plane temperature gradient (see Fig. 1a). An out-of-plane temperature gradient (∇*T*_*z*_) is applied along the *z*-axis using a ceramic heater and a source meter (Keithley-2602A), whereas the temperatures at the hot (bottom) and cold (top) ends were estimated using individual Cernox temperature sensors, as shown in Fig. 1a. Figure 1b shows the thermographic image of a sample to confirm the uniform out-of-plane thermal gradient. The transverse output Nernst voltage i.e. the vortex-Nernst signal (VNE), i.e. *V*_VN_ for NbN thin films and the combination of *V*_VN_, ISHE (*V*_ISHE_) and anomalous Nernst effect (*V*_ANE_) for NbN/Fe bilayers were measured by nanovoltmeter. Figure 1c shows the schematic configuration for the measurement of the vortex Nernst effect generated in NbN and Fig. 1d shows the schematic configuration for the detection of VNE, ISHE and anomalous Nernst effect (ANE) voltage generated in the NbN/Fe bilayer. A PPMS cryostat was used to obtain temperature and magnetic field variations.

## Data Availability

The datasets used and/or analysed during the current study available from the corresponding author on reasonable request.
